# Semiology of Functional Seizures: Sex‐Related Differences

**DOI:** 10.1002/brb3.71610

**Published:** 2026-07-08

**Authors:** Ronen Spierer, Lior Yavetz, Moshe Herskovitz

**Affiliations:** ^1^ Department of Neurology Lady Davis Carmel Medical Center Haifa Israel; ^2^ Rappaport Faculty of Medicine Technion – Israel Institute of Technology Haifa Israel; ^3^ Department of Neurology Rambam Health Care Campus Haifa Israel

**Keywords:** functional seizures, gender, PNES, psychogenic nonepileptic seizures, semiology, sex

## Abstract

**Background:**

While functional seizures (FSs) are far more common among women, previous studies found only a few differences between the two sex groups in relation to semiological characteristics.

**Purpose:**

To compare motor FS semiology between men and women.

**Methods:**

Thirty consecutive females and 28 consecutive males who underwent video‐electroencephalogram and were diagnosed with FSs (without akinetic/dialeptic seizures) were included in this study. Semiology was classified into generalized motor, subjective symptoms, and focal motor. In addition, the involvement of limbs, head, face, and pelvis, as well as the presence of hyperventilation, crying, and pre‐ictal freeze, was compared in occurrence between females and males. A *p*‐value of less than 0.05 was considered significant.

**Results:**

No significant difference was found in the duration of seizures. Also, age at admission and duration of disease did not differ significantly between the groups. Crying and pre‐ictal freeze were significantly more prevalent among females.

**Conclusion:**

Crying and pre‐ictal freeze are more common among females. These findings are consistent with sex‐related differences in trauma‐related defensive responses within the freeze–discharge framework of FSs.

## Graphical abstract

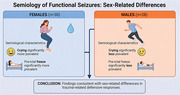

## 1 Introduction

Functional seizures (FSs), also referred to as psychogenic nonepileptic seizures or dissociative seizures, are consistently reported across studies to be far more common among women than men.

According to a review by Fiszman et al. (2004), the rates of previous trauma among FS patients are very high (44%–100%). Dworetzky and Baslet (2017) claimed that psychiatric risk factors, including posttraumatic stress disorder (PTSD), are more prevalent among females within the FS patient population.

In our previous study, we showed that most FSs were preceded by an inactivity phase, leading to the conclusion that FSs are usually a freeze–discharge reaction (Schneider et al. 2022). We proposed that patients re‐experience a trigger associated with the trauma, evoking a defensive freeze response, while the subsequent ictal phase represents the discharge of this state and may serve to relieve physical and mental stress. Accordingly, if PTSD is indeed more common among females with FSs, sex differences in the presence of observable freezing might be expected.

Some theories propose that FSs can reflect unresolved psychological trauma through bodily symbolism. In this view, the body may express traumatic experiences through symbolic movements during the seizure. For example, in individuals with a history of sexual abuse, pelvic or thrusting movements during an episode have been interpreted by some clinicians as a symbolic somatic expression or reenactment of the traumatic experience (Beghi et al. 2017; Bowman 1993; Devinsky 1998).

The purpose of the current study was to compare the semiology of FSs in women and men. By doing so, we can test whether sex differences are present in the occurrence of observable pre‐ictal freezing and in trauma‐related symbolic motor behaviors during the seizure.

## 2 Methods

### 2.1 Patients and Data Collection

The medical records of adult patients admitted to the long‐term video‐electroencephalogram monitoring (LTVEM) unit at Rambam Health Care Campus were reviewed retrospectively. The study included 30 consecutive female patients and 28 consecutive male patients diagnosed with FSs presenting exclusively with motor manifestations. Because the study hypotheses concerned observable motor and pre‐motor phenomena, including pre‐ictal freezing, motor expression, and body region involvement, we restricted the sample a priori to patients with FSs presenting with motor manifestations. Patients with coexisting epileptic seizures and FSs were excluded.

The diagnosis of FSs was made when a typical event occurred without apparent paroxysmal electroencephalogram changes before, during, or after the event. The diagnosis of FSs was made by a neurologist with subspecialty training in epileptology. All FS events occurred spontaneously without the use of induction methods. It is important to note that during LTVEM, we check with the patient and his family members that the events occurring during LTVEM are the typical events occurring at home. We also made sure that the seizure could be visualized from beginning to end within the camera field and that the seizure had a clear motor manifestation. In cases where more than two seizures occurred during monitoring, the analysis was performed on the first two seizures of the patient.

LTVEM was performed using a Natus EMU40EX Wireless LTM amplifier with a high‐definition camera. Electroencephalogram electrodes were placed according to the 10–20 system.

The following baseline and clinical characteristics were collected: age at symptom onset, age at monitoring, duration of disease (defined as the interval from symptom onset to monitoring), duration of individual recorded seizures, and duration of monitoring.

A thorough video analysis was performed. The involvement of specific body regions during seizures (limbs, pelvis, face, and head) was documented as present or absent. Hyperventilation and crying during seizures, as well as freezing before the seizure onset, were likewise evaluated and classified as present or absent. Pre‐ictal freezing was defined as in our previous study: the patient is lying on their back with eyes open or closed and not engaged in any other activity (Schneider et al. 2022). In our previous study, this behavioral pattern was systematically compared between patients with FSs and those with epileptic seizures undergoing the same LTVEM protocol and was found in the majority of FS events but rarely before epileptic seizures, supporting its validity as a specific pre‐ictal FS phenomenon. Phenomenologically, the pre‐ictal freeze is characterized by a behavioral transition from prior activity to stillness occurring in the minutes immediately before seizure onset, often accompanied by a fixed or vacant gaze, qualitatively distinct from casual resting. Responsiveness during the freezing phase was not formally tested with a standardized protocol.

In addition to collecting data on individual semiological features, the seizure descriptions were used to classify the FSs into one of three classes. At least 15 classification systems were suggested for FS semiology (Asadi‐Pooya 2019; Dhiman et al. 2013; Garg et al. 2021). We chose the one that was suggested by Magaudda et al. (2016) and later refined by Asadi‐Pooya et al. (2016), which included generalized motor seizures, focal motor seizures, akinetic seizures, and subjective symptom seizures. As we defined a priori, with the inclusion of only motor FSs, we did not use the category of akinetic seizures. However, in accordance with Asadi‐Pooya et al.’s classification, seizures with subjective symptoms were retained when they were accompanied by permitted minor motor manifestations, such as eyelid myoclonia and/or minor distal limb tremors.

### 2.2 Ethics

All data for the study were collected and analyzed in accordance with the Declaration of Helsinki and were approved by the Institutional Review Board of Rambam Medical Center.

### 2.3 Statistical Analysis

Statistical analyses were performed using SPSS software (IBM, Chicago, USA). Continuous variables are presented as medians and were compared using the Mann–Whitney *U* test. Categorical variables are presented as percentages and were compared using the *χ* ^2^ test. Since this is an exploratory study, no adjustments were made for multiple comparisons.

We also performed a post hoc multivariable analysis for semiological variables that differed significantly between females and males in univariate analysis, with sex as the independent variable of interest and adjustment for age at monitoring. Variables with sparse event counts or unstable model estimates were not included in the multivariable analysis.

All tests performed were two‐tailed, and statistical significance was defined as *p*‐value <0.05.

This is an exploratory, retrospective, consecutive patient study; accordingly, no formal a priori power calculation was performed. In addition, no adjustments were made for multiple comparisons in keeping with the exploratory design. Therefore, our findings should be regarded as hypothesis‐generating.

## 3 Results

Between March 1, 2018, and December 31, 2024, a total of 623 video‐EEG recordings were performed at our center. Of these, 212 patients were diagnosed with FSs. Thirty consecutive female patients and 28 consecutive male patients were included in our analysis.

Age at symptom onset, age at monitoring, and duration of disease did not differ significantly between males and females. Overall, all demographic and LTVEM characteristics were similar between the two sex groups (Table 1).

**TABLE 1:** Demographic and clinical characteristics by sex.

	Female (*n* = 30)	Male (*n* = 28)	*p*‐value
Age at symptoms onset (years), median (IQR)^a^	27 (19.25, 38)	25.5 (19, 40)	0.642
Age at monitoring (years), median (IQR)	36 (25.25, 51)	28 (22, 42)	0.259
Duration of disease (years), median (IQR)^a^	3 (1, 10)	1 (0.5, 6)	0.155
Duration of seizures (min), median (IQR)	7.5 (5.25, 18.75)	9 (4, 16)	0.596
Duration of monitoring (days), median (IQR)	5 (3.25, 5)	4 (3, 8)	0.918

Abbreviation: IQR, interquartile range.

^a^ Data are available for 51 patients.

Body regions involved during seizures did not show significant sex‐related differences (Table 2). Similarly, seizure semiological type did not differ significantly between males and females (Table 3). In contrast, specific ictal behavioral manifestations demonstrated sex‐related variability: crying (*p* = 0.045) and pre‐ictal freeze (*p* = 0.018) were significantly more common among female patients. In the multivariable analysis, including sex, age, and freeze, sex remained significantly associated with freeze (*p* = 0.025).

**TABLE 2:** Frequencies of regions involved by sex.

	Female (*n* = 30)	Male (*n* = 28)	*p*‐value
Limbs, *n* (%)	27 (90.0)	23 (82.1)	0.386
Pelvis, *n* (%)	3 (10.0)	3 (10.7)	0.929
Face, *n* (%)	7 (23.3)	8 (29.6)	0.590
Head, *n* (%)	13 (43.3)	14 (50.0)	0.611

**TABLE 3:** Frequencies of other semiological signs by sex.

	Female (*n* = 30)	Male (*n* = 28)	*p*‐value
Type, *n* (%)			0.958
Generalized motor	14 (46.7)	12 (42.9)	
Subjective symptoms	8 (26.7)	8 (28.6)	
Focal motor	8 (26.7)	8 (28.6)	
Hyperventilation, *n* (%)	6 (20.0)	9 (32.1)	0.291
Crying, *n* (%)	4 (13.3)	0 (0.0)	0.045
Freeze, *n* (%)	23 (76.7)	13 (46.4)	0.018

## 4 Discussion

This study was conducted to examine whether FSs are exhibited differently between males and females. We did not observe any significant baseline differences between women and men with FSs. As to semiological sex‐related differences, both crying and freezing before the seizure's onset were significantly more common among females.

To begin with, the high proportion of pre‐ictal freezing is consistent with our previous hypothesis of FSs as an activation of inherent hardwired behavior (reflecting an underlying trauma‐related response) (Schneider et al. 2022). Moreover, the higher occurrence among females may shed light on the underlying pathophysiological mechanisms of FSs. While one study reported no significant sex differences in PTSD comorbidity among patients with FSs (Libbon et al. 2020), another found sex‐related differences in predisposing factors, that is, sexual abuse in females and learning disability in males, although this was observed in an adolescent population (Yeom et al. 2022). Recently, Vilyte et al. (2026) reported that a history of sexual abuse was more common among females. Our findings appear to be more consistent with the latter observations and with others that found a history of sexual and physical abuse to be more prevalent among females (Asadi‐Pooya et al. 2019; Oto et al. 2005; Thomas et al. 2013). Taken together, the high prevalence of pre‐ictal freezing, its greater occurrence among females, and previous evidence that females with FSs more often report trauma‐related backgrounds support the view of FSs as a freeze–discharge response. However, no individual‐level data on past traumatic experiences or PTSD were collected in this study, and therefore, the proposed link between pre‐ictal freezing and trauma remains speculative.

As for body regions involved and semiological type, we did not detect any significant sex‐related differences, consistent with previous studies. As detailed in Table 4, several studies did not detect any differences (Gale et al. 2015; Thomas et al. 2013), while others found only minimal semiological differences (Asadi‐Pooya et al., 2013, 2019; Korucuk et al. 2018; Oto et al. 2005; Ture et al. 2019; Vilyte et al. 2026). Surprisingly, Türe et al. found that opisthotonic and pelvic thrust motion were more common among men (Ture et al. 2019). This finding is unexpected, as such movements have sometimes been interpreted as reflecting trauma‐related or sexually themed symbolic motor expressions. In light of theories suggesting that FSs may represent bodily expressions of unresolved psychological trauma, including symbolic reenactment of traumatic experiences, these movements might have been anticipated to occur more frequently in females.

**TABLE 4:** Results from previous studies focusing on semiological sex‐related differences.

Study	Country	Females:males	Semiological differences	Other findings
Oto et al. (2005)	United Kingdom	117:43	Crying was more common among females.	Females had an earlier age of onset. Males had higher rates of unemployment. Males had higher rates of predisposing factors for epilepsy. Females had higher rates of self‐harm. Carers of females were less likely to reject the diagnosis. History of sexual abuse was more common among females.
Asadi‐Pooya et al. (2013)	Iran	129:59	Fine shaking movements were more common among females. Aura was more common among females.	History of significant head trauma was more common among males. Having a medical comorbidity was more common among females.
Thomas et al. (2013)	Lebanon	59:27		Psychiatric comorbidity was more common among females. History of sexual abuse was more common among females. History of physical abuse was more common among females. Chronic pain was more common among females.
Gale et al. (2015)	United States	147:58		Females had higher scores on the physiological depression scale. Males had higher scores on the Borderline Features subscales, including affective instability and self‐harm, and on the Antisocial Features scale.
Korucuk et al. (2018)	Turkey	31:10	Ictal duration was longer for females. Crying was more common among females.	Age at onset was earlier for males.
Asadi‐Pooya et al. (2019)	Iran, United States, Canada, Brazil, Argentina, and Venezuela.	305:146	Aura was more common among females.	Age at onset was earlier for males. Age at monitoring was earlier for males. History of sexual abuse was more common among females. History of physical abuse was more common among females. History of family dysfunction was more common among females.
Ture et al. (2019)	Turkey	92:63	Major motor activity was more common among males. Opisthotonic and pelvic thrust motions were more common among males. Lateralizing motion was more common among males. Crying was more common among females.	
Vilyte et al. (2026)	South Africa	279:93	Reported loss of consciousness was more common among males. Side‐to‐side head movements were more common among females.	History of any psychological trauma/stressor (especially sexual abuse) was more common among females. Use of anti‐seizure medications was more common among males.

Observations in animal models, where the transition from a freezing state to active movement is characterized as a discharge reaction, suggest that the seizure‐like tremors or movements are interpreted not as symbolic gestures but as a physiological attempt to complete the interrupted flight response and return the autonomic nervous system to homeostasis (Schneider et al. 2022). If FS indeed represents a similar biological release of a pent‐up survival act, the lack of sex‐specific symbolic movements in our study and the higher prevalence of pre‐ictal freezing in females suggest that these episodes are rooted in hardwired evolutionary defenses rather than elective symbolic reenactment.

In our study, four of the females presented with ictal crying, compared to none of the males. This finding is in agreement with the studies of Korucuk et al. and Türe et al., which found this feature present in females but not in males (Korucuk et al. 2018; Ture et al. 2019), and with the study by Oto et al. that found ictal crying to be more common among women (Oto et al. 2005). In contrast, Bergen and Ristanovic presented a series of 10 FS patients with ictal crying, with half of them being males (Bergen and Ristanovic 1993). Moreover, other reports found the frequency differences in crying between female and male FS patients insignificant (Asadi‐Pooya et al. 2019; Thomas et al. 2013; Walczak and Bogolioubov 1996). These reports cannot be overlooked, but still, it is most probable that crying during an FS is a female characteristic, as crying in general is more common among women (Becht and Vingerhoets 2002).

Although not part of our analysis, the use of anti‐seizure medications (ASMs) may also be relevant to mention. In previous studies, women and men were found to be equally likely to be treated with ASMs (Asadi‐Pooya and Bahrami 2019; Spierer and Herskovitz 2024). In contrast, Vilyte et al. (2026) found that in one of two studied groups, males were more likely to report a history of failed ASMs.

This study has several limitations. One obvious limitation is the study's retrospective design. In addition, only one evaluator analyzed the videos, and blinding to sex was impractical, potentially introducing measurement bias. Furthermore, the study was conducted at a single center, which may limit the generalizability of the findings. Also, this study was exploratory; therefore, all findings should be regarded as hypothesis‐generating and requiring replication in confirmatory studies. No individual‐level data on traumatic experiences, PTSD diagnosis, dissociative symptoms, or abuse history were collected; consequently, the proposed link between pre‐ictal freezing and trauma‐related pathophysiology remains speculative and requires direct empirical testing in prospective studies incorporating structured psychiatric assessments. However, the fact that semiological analysis was performed without access to etiological data means that the observed behavioral patterns reflect objective video findings rather than a narrative‐driven assessment.

Additionally, responsiveness during the pre‐ictal freeze phase was not formally tested with a standardized protocol in all cases, and the behavioral distinction from relaxed inactivity relied on expert video review. Finally, the restriction to motor FSs means that these findings cannot be generalized to the full spectrum of FSs, including akinetic and dialeptic forms.

In conclusion, we did not find a major difference in FS semiology between the two sexes. However, two characteristics—crying and pre‐ictal freezing—were more common among females with FSs, with the latter being a novel finding. Our findings, together with the existing literature, exclude the possibility of symbolism (“semiology shaped by trauma”) in FS semiology. Instead, it aligns with the possibility of FSs being associated with PTSD in women.

## Author Contributions

**Ronen Spierer**: investigation, writing – review and editing, methodology, validation, visualization, formal analysis, project administration, supervision, writing – original draft. **Lior Yavetz**: investigation, writing – original draft, writing – review and editing, data curation. **Moshe Herskovitz**: conceptualization, investigation, writing – original draft, writing – review and editing, validation, methodology, data curation, supervision, project administration.

## Funding

The authors have nothing to report.

## Conflicts of Interest

The authors declare no conflicts of interest.

## Data Availability

Anonymized data will be shared upon request from any qualified investigator.
